# The social stigma of infertile women in Zhejiang Province, China: a questionnaire-based study

**DOI:** 10.1186/s12905-021-01246-z

**Published:** 2021-03-05

**Authors:** Fang Zhang, Yan Lv, Yanting Wang, Xuzhen Cheng, Yuying Yan, Ye Zhang, Yue Wang

**Affiliations:** 1 Department of Assisted Reproduction, Women’s Hospital, Zhejiang University School of Medicine, Hangzhou, Zhejiang China; 2 Sir Run Run Shaw Hospital, Zhejiang University School of Medicine, Hangzhou, Zhejiang China; 3 Institute of Translational Medicine, Zhejiang University School of Medicine, Hangzhou, Zhejiang China; 4 Ambulatory Surgery Center, Women’s Hospital, Zhejiang University School of Medicine, No. 1 Xueshi Road, Hangzhou, Zhejiang Province China

**Keywords:** ISS, Female infertility, Stigma, Questionnaire

## Abstract

**Background:**

Infertile women often face stigmatization worldwide. This study aimed to investigate the stigma against infertile women in China and to analyze its influencing factors.

**Methods:**

Of 270 women who were randomly selected from patients receiving adjuvant fertility treatment in Zhejiang Province, China, 254 successfully completed the general information questionnaire, disease information questionnaire, and Chinese version of the infertility stigma scale (ISS). The ISS contained 27 positively worded items, each of which was graded on a 5-point Likert-type scale.

**Results:**

The total stigma score of female infertility patients was 66.39 ± 21.96. By dividing the number of items, the average score for each ISS item was 2.13 ± 0.81, indicating the presence of stigma. Among the four ISS factors, the social withdrawal score was the highest (2.64 ± 1.05), whereas the family stigma score was the lowest (1.88 ± 0.88). Multiple stepwise regression analysis further revealed that the duration of infertility and monthly income were important predictors of the stigma of infertile women.

**Conclusions:**

Infertile women experience moderate to high levels of stigma in Zhejiang, China. Thus, supportive psychological interventions and public education are required to change patients’ cognition and assist patients in coping with negative experiences.

**Supplementary Information:**

The online version contains supplementary material available at 10.1186/s12905-021-01246-z.

## Background

Infertility has been defined as the failure to conceive after one year of regular unprotected sexual intercourse [1]. There are two types of infertility, primary and secondary infertility. The former refers to the infertility of couples who have never conceived, while the latter refers to the infertility of couples who have conceived at least once before. More than 70 million couples suffer from infertility worldwide. The incidence of female infertility is 6.9–9.3% in developing countries but is 3.5–16.7% in developed countries [2–5]. In China, infertility affects approximately 15% of the birth population, namely, over 50 million infertile patients according to the 2014 National Conference on Infertility [6].

Infertile women experience the negative consequences of childlessness to a greater degree in developing countries than in developed societies [2]. Globally, childlessness creates problems for couples, especially for women, who are generally blamed for couples’ infertility and suffer personal grief and frustration, social stigma, rejection and serious economic deprivation [7]. In 2007, Slade et al. investigated the stigma level of 87 infertile women who sought treatment in the UK [8]. The stigma score was 22.30 ± 9.93 according to the Stigma Consciousness Questionnaire, indicating a high level of stigma. In 2015, Jansen et al. collected posts and comments from 432 infertile American females who inquired about treatment on online forums [9]. These patients were found to have a strong sense of stigma when others talked about their infertility, and therefore felt social withdrawal. In Cameroon, stigmatization is also an important reason for divorce among the Bangangte tribes, causing women to lose their access to land distributed by their husbands [10]. Similarly, 64% of infertile women felt stigmatized in southern Ghana [11].

In China, women usually have relatively high family decision-making power [12]. Due to the great importance attached to fertility and childbearing ability in China, however, there is a tendency in society to perceive that “Childlessness is always the woman’s fault.” Taking Zhejiang Province as an example, where the economy is prosperous, women usually receive a good education [12, 13]. Because of the traditional gender perception and social culture, infertility still leads to stigmatization and discrimination, as well as potentially unstable marriages, which brings women huge mental pressure [14]. Unfortunately, we were unable to find further evidence to support the stigma associated with infertility in this cultural context. Moreover, infertile women need to spend a greater amount of time and money on treatment, which causes additional psychological disorders, such as depression and anxiety.

Most of the negative emotions caused by infertility have been significantly related to stigma [15]. Stigma may lower self-esteem and self-efficacy in infertile women [16] and is also associated with increased distress, low social support and low social status [15]. It is therefore important for medical caregivers to determine and deal with the psychological aspects of infertility.

Many of the current self-reporting measures that evaluate infertility stigma are generic measurements, including the stigma consciousness questionnaire and perceived stigma scale [8, 11]. Generic measurements clearly lack quantitative sensitivity to patients coping with infertility. Thus, Fu et al. developed an infertility stigma scale (ISS) and applied it to infertile women in Hunan Province, China to assess their perceived stigma and self-stigmatization [15]. However, although they designed the questionnaire and proved its validity and reliability, they did not describe the detailed extent of stigma of infertile women in China.

In the present study, we aimed to determine the effects of infertility on the internal stigmatization experienced by a group of infertile women who were undergoing treatment for assisted reproductive technology. In addition, we aimed to underline the necessity of psychological support for infertile women, which is of equal importance to medical treatment.

## Methods

### Design and setting

The present questionnaire-based study was conducted in Women’s Hospital, Zhejiang University School of Medicine, Hangzhou, China between 1 Oct 2017 and 31 Mar 2018.

### Participants and recruitment

A population that includes a minimum number of individuals of five to 10 times higher than the number of scale items is required [15]. As the ISS contains 27 items, a total of 270 women meeting the study criteria were required for the present study. The inclusion criteria involved being a primary school graduate at a minimum, receiving infertility treatment, and agreeing to participate in the study. The exclusion criteria included: (i) severe heart, brain or kidney diseases; (ii) severe mental disorders; (iii) genetic diseases; and (iv) severe organic diseases in the reproductive or endocrine system.

We selected 298 female infertility patients among the women who were accepting treatment for assisted reproductive technology (ART) in the hospital. Twenty-eight refused to participate, with the remaining 270 agreeing to participate in the study. Finally, 254 respondents successfully met the inclusion criteria.

### Questionnaire design and measurement

After reviewing the literature and consulting the relevant experts, we designed a general information questionnaire and a disease information questionnaire (Additional file 1: Table 1 Part A). The former mainly investigated age, education level, income, family type, work status, medical payment status and marital status. The latter mainly investigated previous pregnancy, planned fetal number, infertility cause, and duration of infertility.

The Chinese ISS questionnaire was designed by Fu et al. (Additional file 1: Table 1 Part B). and contains 27 items divided into four factors: self-devaluation (7 items), social withdrawal (5 items), public stigma (9 items), and family stigma (6 items) [15]. The responses to each item were based on a 5-point Likert scale (1 = completely not agree, 2 = not agree, 3 = not sure, 4 = agree, 5 = completely agree). All items were positively worded. Thus, the total score ranged between 27 and 135, with a higher score representing a higher level of stigma.

Before the investigation was carried out, four nurses with at least 3 years of working experience were recruited and trained as investigators. The investigators explained to the participants the purpose, significance and method of filling out the questionnaires and explained the contents of the questionnaire if the participants did not understand. The participants were required to complete the general information questionnaire, disease information questionnaire, and ISS questionnaire independently.

### Statistical analysis

Data analysis was performed by using SPSS (version 18; SPSS Inc., Chicago, IL). Numbers and percentages were utilized to describe discrete variables, and means and standard deviations were utilized to describe discrete variables. Cronbach's α coefficients were calculated to evaluate the internal consistency of the scales within and between the four ISS factors. Student’s t test was applied to assess the significance of each stigma factor. Student’s t test, one-way ANOVA (plus Tukey post hoc analysis) and multiple stepwise regression analysis were performed to identify factors affecting stigma. The value 0.05 was set as the threshold of significance.

### Ethics

The study was approved by the Ethics Committee of Women’s Hospital in the Zhejiang University School of Medicine (project 20170186). Furthermore, verbal and written consent was received from the women who agreed to take part in the research.

## Results

### General information on the female infertility patients

The characteristics of the participants are listed in Table 1. All respondents were married and had no children. The majority of them were 26–45 years old, and their duration of infertility was over three years. Approximately two-thirds of the respondents were employed, had conceived before, and had no medical insurance. Tubal problem was the major cause of infertility.

**Table 1:** Sociodemographic and clinical characteristics of the respondents and their association with stigma (Hangzhou, China; 2017–2018)

Variable	Cases	Percentage (%)	Stigma score	*P* value
Age (year)				0.61
20–25	11	4.33	52.27 ± 30.00	
26–35	164	64.57	55.91 ± 22.33	
36–45	73	28.74	58.64 ± 19.1	
46–50	6	2.36	49.5 ± 28.51	
Education level				< 0.01
Elementary school	5	1.97	81.4 ± 15.96	
Junior high school	62	24.41	63.34 ± 21.10	
High school	40	15.75	53.95 ± 24.94	
Secondary school	21	8.27	56.24 ± 16.61	
College	47	18.5	53.17 ± 22.88	
Bachelor’s degree	69	27.17	52.88 ± 20.6	
Master’s degree and above	10	3.94	50.1 ± 16.86	
Monthly Income (Yuan)				< 0.01
< 3000	33	12.99	71.97 ± 20.39	
3000–6000	88	34.64	55.21 ± 22.55	
6000–10,000	77	30.31	55.27 ± 20.92	
> 10,000	56	22.05	50.63 ± 20.19	
Whether the patient is an only child				0.95
Yes	60	23.62	56.23 ± 20.70	
No	194	76.38	56.43 ± 22.33	
Whether the husband is an only child				0.26
Yes	90	35.43	54.28 ± 21.78	
No	164	64.57	57.54 ± 21.97	
Family types				0.41
Living with the husband only	114	44.88	55.93 ± 21.02	
Living with the husband and parents-in-law	103	40.55	57.83 ± 23.07	
Living with the husband and own parents	23	9.06	49.96 ± 18.00	
Living with the husband, his brother’s family and parents-in-law	14	5.51	60.14 ± 27.27	
Work status				< 0.01
Yes	163	64.17	53.27 ± 20.29	
No	91	35.83	61.67 ± 23.80	
Medical payment status				0.03
Insurance-paid	84	33.07	52.13 ± 21.34	
Self-paid	170	66.93	58.51 ± 22.02	
Marital status				0.24
First marriage	220	86.61	55.87 ± 22.07	
Remarriage	34	13.39	60.59 ± 20.45	
Previous pregnancy				0.72
Yes	164	64.57	56.13 ± 21.52	
No	90	35.43	57.18 ± 22.63	
Fetal number				0.48
First	181	71.26	56.93 ± 22.67	
Second	73	28.74	54.75 ± 20.15	
Infertility cause				0.047
Ovulation disorder	17	6.69	50.41 ± 18.67	
Tubal factor	145	57.09	50.52 ± 22.12	
Endometrial factor	49	19.29	52.63 ± 23.26	
Unknown factor	43	16.93	59.86 ± 19.26	
Duration of infertility (years)				0.017
< 3	25	9.84	48.45 ± 19.18	
3–5	112	44.09	53.97 ± 20.19	
> 5	117	46.06	60.48 ± 23.64	

### The status of stigma in female infertility patients

The questionnaire results showed that the total stigma score of the respondents was 66.39 ± 21.96. The 25% percentile, median, and 75% percentile were 49, 66 and 80, respectively. The skewness was 0.25. The average item score for the total ISS was 2.13 ± 0.81, while the average item scores for the factors self-devaluation, social withdrawal, public stigma, and family stigma were 2.11 ± 0.91, 2.64 ± 1.05, 1.91 ± 0.88, and 1.88 ± 0.88, respectively. Compared with the neutral value of 2.5, social withdrawal showed a significantly higher value, whereas the other three factors showed significantly lower values. Thus, social withdrawal was remarkably present among the respondents.

The Cronbach’s α coefficient represents an estimation method of internal consistency used for Likert‐type scales. In the present study, the Cronbach’s α coefficient of the total scale was determined to be 0.968, that of the factor self‐devaluation was 0.910, that of the factor social withdrawal was 0.877, that of the factor public stigma was 0.961, and that of the factor family stigma was 0.82. Accordingly, the internal validity of the scale was quite good.

### Variables associated with stigma in female infertility patients

Next, we tested the association between stigma and the variables investigated in the general information questionnaire and disease information questionnaire. Taking the total ISS score as the observation index, a one-way ANOVA or Student’s t test was performed, which showed that the total ISS score was statistically associated with education level, monthly income, work status, medical insurance coverage, infertility factors and duration of the infertility period (*P* < 0.01; Table 1).

Based on the above, a multiple stepwise regression was further carried out; all variables with a *P* value < 0.05 were included in the analysis. As a result, monthly income and the duration of infertility were retained in the equation for the multivariate stepwise regression. As shown in Table 2, monthly income had a negative predictive effect on the total ISS score, indicating that higher monthly income was associated with a lower degree of stigma. In contrast, the duration of infertility had a positive predictive effect on the total ISS score, indicating that longer infertility was associated with a greater degree of stigma.

**Table 2:** A multiple stepwise regression analysis of the factors affecting stigma in infertile women (Hangzhou, China; 2017–2018)

Variables	Regression coefficients	Standard error	Standardization coefficient	*P* value	95% Confidence interval
Monthly income	− 4.61	1.45	− 0.20	0.00	− 7.48 to − 1.74
Duration of infertility	5.98	2.19	0.17	0.00	1.66 to 10.30

## Discussion

### The status of stigma among female infertility patients

The present study shows that infertile women generally have a sense of stigmatization, particularly for the factor of social withdrawal. These results are similar to the results of Donkor et al. and Hollos et al. [11, 17]. In 2015, Jansen et al. observed 432 infertile women seeking treatment in the United States through online forums and pointed out that social withdrawal is one of the mechanisms used by infertile women to cope with stigma [9]. According to the social culture and traditional concepts in China, family succession has become an ethical issue in the public's mind and a generally accepted fertility value [18]. Many female infertility patients themselves have a strong desire to conceive and are more sensitive to words such as "pregnancy" and "children." Most of their peers already have children, and they feel different from others. They feel upset when asked about their children or when they face comments from others. They feel "pathetic" in the eyes of others and are considered a "joke." To escape these inner thoughts and avoid embarrassing situations, they are unwilling to go to parties with friends who have children, participate in activities, or contact people, which might result in social withdrawal.

### Factors affecting the stigma of female infertility patients

According to the one-way ANOVA, the affecting factors mainly include education level, monthly income, occupation, medical coverage, infertility causes and the duration of the infertility period. Regarding these factors, we have the following findings. First, women holding a Master’s degree or higher experienced less stigmatization. Donkor et al. also found that tertiary education and higher social status were mediating factors in reducing a woman's perceived stigma [11]. This may be because women with higher educational levels tend to hold relatively stable employment positions and incomes. Their greater access to knowledge and their ability to study, communicate and adjust psychologically may also help them acquire effective treatment measures, relieve themselves from traditional discrimination, and avoid any sense of stigmatization [18, 19].

Second, the employed female patients and patients with high income had a lower sense of stigma. This is probably because these patients tend to devote more time and energy to their careers, recognize ego value, and win respect from others. Thus, work can help distract their attention from infertility.

Third, patients with shorter durations or with medical healthcare insurance had a lower sense of stigmatization. Alhassan and colleagues also pointed out that a longer duration of infertility was positively associated with depression [19]. Due to potential pressure from the surrounding environment and public opinion, a considerable number of infertile women choose to conceal their condition and even refuse to seek medical treatment, which delays their recovery from the disease [8]. Because longer treatment periods or greater numbers of failed treatments cost more money, appropriate medical insurance can effectively alleviate the patients’ financial burdens, thus reducing the degree of stigma.

Fourth, patients with ovulation disorders have the lowest stigma levels, followed by those with tubal infertility. In vitro fertilization for patients with ovulation disorders and tubal infertility, i.e., through embryo transfer, usually has a higher success rate according to data from our reproductive center. In contrast, endometrial and unexplained infertility are more difficult to treat, which explains their higher degree of stigma.

### Advice on clinical care of infertile women

Few studies have been formally conducted on interventions related to stigma among infertile women, but the following approaches have been recommended in the literature [20]. The first approach is professional psychological consultation. Regular consultations can cover medicine as well as daily life, job hunting and other related topics. The consultants must have good insight into the patients’ emotional and psychological changes, understand the patients’ thinking, analyze the causes of stigma, and offer patients with proper psychological guidance and cognitive intervention. During consultation, nurses should play a pivotal role in addressing the stigma of infertility [16]. Both one-on-one and group consultations should be performed because the latter can motivate patient-patient communication and mutual support [20].

The second approach is social support in the community. An important reason for stigma is rejection and discrimination from the outside world and even from relatives and friends. Establishment of a "care-support" education club in the community is recommended [18]. On the one hand, the population can be educated in the club with knowledge of social care, through which the public’s stereotypes can be corrected, and a more tolerant and positive social environment can be created for patients [18]. On the other hand, family members and relatives should be educated to provide patients with more support and care to prevent patients from feeling alienated. This will undoubtedly help alleviate the patients’ stress and improve their self-confidence and self-esteem.

Third, better public medical healthcare should be implemented. The costs of infertility treatment are high because infertility has not yet been covered by medical insurance in most regions of China. Since economic factors are tightly associated with stigma, the establishment of targeted medical insurance plans or expense reimbursement can help patients accept more active interventions, therefore relieving a great burden on patients and their families.

### Limitations

One limitation of the present study is that we only collected data from women who ‘experienced’ stigma, not from others ‘against’ them, which may have caused deviations.

Second, this study did not include stigma against infertile men. The causes of infertility from men and women are roughly half-and-half [21]. In Chinese culture, male factor infertility usually suffers more stigma. Accordingly, infertile men tend to hide the truth, and men who do not know whether they are infertile are not willing to accept physical examination. Thus, infertility tends to be attributed to women by society, regardless of whether women accept it. A lack of investigation of infertile men will aggravate society’s misunderstanding of women with infertility and women’s own suspicion of themselves. This is not good for improving the stigma level of women with infertility.

## Conclusions

In conclusion, infertile women experience moderate to high levels of stigma in Zhejiang, China, which mainly presents as social withdrawal. Thus, supportive psychological interventions and public education are required to change patients’ cognition and assist patients in coping with negative experiences.

## Supplementary Information

**Additional file 1:** Questionnaires of general information, disease information and the Chinese ISS.

## Data Availability

The datasets used and analyzed during the current study are available from the corresponding author on reasonable request.

## Abbreviations

ISS: Infertility Stigma Scale
ANOVA: Analysis of Variance
