# Supplementary material for: The social stigma of infertile women in Zhejiang Province, China: a questionnaire-based study
Source: BMC Womens Health. 2021 Mar 5;21:97. doi: 10.1186/s12905-021-01246-z (PMC7934237; doi:10.1186/s12905-021-01246-z)
Supplement: Supplementary file 1 — Additional file 1: Questionnaires of general information, disease information and the Chinese ISS. [file 12905_2021_1246_MOESM1_ESM.docx]

| **Health Questionnaire** | | | | | | | |
| --- | --- | --- | --- | --- | --- | --- | --- |
| Dear Madam：  In order to better understand the general psychological situation of infertile women, we will collect some information about you, and we hope that you can answer honestly. The information you provided will help medical workers truly understand the infertility, and we will work hard and continuously to reduce negative attitude of the public towards infertility and the stigma of the infertility. You participate in this survey on a voluntary basis, and we will respect you choices. You can also quit the survey at anytime, and your participation will not do you any harm. The survey is anonymous, we will keep the information you provided strictly confidential. Thank you very much for your support and help! | | | | | | | |
| A. Basic information | | | | | | | |
| A1 | Age(Year):  □20-25 □26-35 □36-45 □46-50 | | | | | | |
| A2 | Educational level:  □Never received a normal education □Graduated from elementary school □Graduated from junior high school □Graduated from high school  □Graduated from secondary school □Graduated from college  □Bachelor’s degree □Master’s degree and above | | | | | | |
| A3 | The average monthly incomes of your family（Yuan）：  □<3000 □3000-6000 □6000-10000 □>10000 | | | | | | |
| A4 | Your habitual residence for the past year： District City Province Urban/Rural area | | | | | | |
| A5 | Whether the patient is an only child? | | □Yes □No | | | | |
| A6 | Whether the husband is an only child？ | | □Yes □No | | | | |
| A7 | Family types:  □Living with the husband only  □Living with the husband and parents-in-law  □Living with the husband and own parents  □Living with the husband, his brother’s family and parents-in-law | | | | | | |
| A8 | Work status: | | □Yes □No | | | | |
| A9 | Medical payment status: | | □Insurance-paid □Self-paid | | | | |
| A10 | Martial status: | | □First marriage □Remarriage | | | | |
| A11 | Previous pregnancy: | | □Yes □No | | | | |
| A12 | Fetal number | | □First □Second | | | | |
| A13 | Infertility cause:  □Ovulation disorder □Tubal factor  □Endometrial factor □Unknown factor | | | | | | |
| A14 | Duration of infertility (years):  □<3 □3-5 □>5 | | | | | | |
| B Mental Health: The following is about your psychological activities caused by infertility, please tick the eligible options without worries. | | | | | | | |
| Item | | Totally disagree | | Partially disagree | Uncertain | Partially agree | Totally agree |
| **Self-devaluation** | | | | | | | |
| 1. I feel that I have an unfortunate fate. | | 1 | | 2 | 3 | 4 | 5 |
| 2. I feel that I am a failure to be a woman. | | 1 | | 2 | 3 | 4 | 5 |
| 3. I feel like a burden to my family. | | 1 | | 2 | 3 | 4 | 5 |
| 4. I feel inferior to others because of infertility. | | 1 | | 2 | 3 | 4 | 5 |
| item | | Totally disagree | | Partially disagree | Uncertain | Partially agree | Totally agree |
| 5. I am ashamed of being infertility. | | 1 | | 2 | 3 | 4 | 5 |
| 6. I look down on myself because of infertility. | | 1 | | 2 | 3 | 4 | 5 |
| 7. I feel useless at times. | | 1 | | 2 | 3 | 4 | 5 |
| **Social withdrawal** | | | | | | | |
| 1. I am more sensitive to pregnancy and child because I can’t get pregnant. | | 1 | | 2 | 3 | 4 | 5 |
| 2. I feel embarrassed when being asked something about the kids. | | 1 | | 2 | 3 | 4 | 5 |
| 3. I avoid getting close to people who don’t have fertility problem. | | 1 | | 2 | 3 | 4 | 5 |
| 4. I am unwilling to mention infertility. | | 1 | | 2 | 3 | 4 | 5 |
| 5. I try to conceal my conditions form others. | | 1 | | 2 | 3 | 4 | 5 |
| Public stigma | | | | | | | |
| 1. It is common that people discriminate against infertile women. | | 1 | | 2 | 3 | 4 | 5 |
| 2. I dare not to make new friends lest they find out that I have infertility. | | 1 | | 2 | 3 | 4 | 5 |
| 3. I worry that people may stay away from me when they find out I have infertility. | | 1 | | 2 | 3 | 4 | 5 |
| 4. I worry that people may look down on me when they find out I have infertility. | | 1 | | 2 | 3 | 4 | 5 |
| 5. I worry that people may laugh at me when they find out I have infertility. | | 1 | | 2 | 3 | 4 | 5 |
| 6. I feel like a freak (an incomplete woman) in the eyes of others. | | 1 | | 2 | 3 | 4 | 5 |
| 7. I feel that people judge me behind my back because of infertility. | | 1 | | 2 | 3 | 4 | 5 |
| 8. I feel that people around look down on me because of infertility. | | 1 | | 2 | 3 | 4 | 5 |
| 9. I feel that people view me differently because of infertility. | | 1 | | 2 | 3 | 4 | 5 |
| **Family stigma** | | | | | | | |
| 1. Having infertility has spoiled my life. | | 1 | | 2 | 3 | 4 | 5 |
| 2. I worry that the relationship with my husband would be worse. | | 1 | | 2 | 3 | 4 | 5 |
| 3. I am afraid my husband would divorce with me. | | 1 | | 2 | 3 | 4 | 5 |
| 4. I feel that my family does not take care for me as much as before. | | 1 | | 2 | 3 | 4 | 5 |
| 5. My family, especially my mother-in-law, were always trying to make trouble for me. | | 1 | | 2 | 3 | 4 | 5 |
| 6. I am afraid my remarriage would be affected, once people know my situation. | | 1 | | 2 | 3 | 4 | 5 |
|  | | | | | | | |
